# Hepatitis E virus infections among patients with acute febrile jaundice in two regions of Cameroon: First molecular characterization of hepatitis E virus genotype 4

**DOI:** 10.1371/journal.pone.0298723

**Published:** 2024-02-12

**Authors:** Abdou Fatawou Modiyinji, Lange Tchamba Amorgathe Tankeu, Chavely Gwladys Monamele, Moise Henri Yifomnjou Moumbeket, Paul Alain Tagnouokam Ngoupo, Huguette Tchetgna Simo, Abanda Njei Ngu, Kazanji Mirdad, Richard Njouom

**Affiliations:** Virology Unit, Centre Pasteur du Cameroun, Yaoundé, Cameroon; CEA, FRANCE

## Abstract

**Background:**

Febrile jaundice is a common indicator of certain infectious diseases, including hepatitis E. In Cameroon, the yellow fever virus is the only pathogen that is monitored in patients who present with this symptom. However, more than 90% of the samples received as part of this surveillance are negative for yellow fever. This study aimed to describe the prevalence and hepatitis E virus (HEV) genotype among yellow fever-negative patients in the Far North and West regions of Cameroon.

**Methods:**

In a cross-sectional study, yellow fever surveillance-negative samples collected between January 2021 and January 2023 were retrospectively analyzed. Anti-HEV IgM and IgG antibodies were tested using commercially available ELISA kits. Anti-HEV IgM and/or IgG positive samples were tested for HEV RNA by real-time RT-PCR, followed by nested RT-PCR, sequencing and phylogenetic analysis.

**Results:**

Overall, 121 of the 543 samples (22.3%, 95% CI: 19.0% - 26.0%) were positive for at least one anti-HEV marker. Amongst these, 8.1% (44/543) were positive for anti-HEV IgM, 5.9% (32/543) for anti-HEV IgG, and 8.3% (45/544) for both markers. A total of 15.2% (12/79) samples were positive for HEV RNA real-time RT-PCR and 8 samples were positive for HEV RNA by nested RT-PCR. Phylogenetic analysis showed that the retrieved sequences clustered within HEV genotypes/subtypes 1/1e, 3/3f and 4/4b.

**Conclusion:**

Our results showed that HEV is one of the causes of acute febrile jaundice in patients enrolled in the yellow fever surveillance program in two regions of Cameroon. We described the circulation of three HEV genotypes, including two zoonotic genotypes. Further studies will be important to elucidate the transmission routes of these zoonotic HEV genotypes to humans in Cameroon.

## Introduction

Hepatitis E virus (HEV) is a known cause of human viral hepatitis and is probably the most common cause of acute hepatitis and jaundice worlwide [1]. Approximately 20 million HEV infections occur each year causing 3 million cases of acute hepatitis and 44,000 deaths, according to the World Health Organization (WHO) [2]. HEV infection is usually acute and self-limiting in immunocompetent individuals. However, chronic HEV infection can occur in immunocompromised patients [3]. The typical symptoms of hepatitis E include fatigue, loss of appetite, abdominal pain, nausea, fever, and jaundice [4].

HEV is a single-stranded, positive-sense RNA virus with a genome size of approximately 7.2 kb. The genome consists of three partially overlapping open reading frames (ORF1, ORF2 and ORF3). However, a fourth open reading frame (ORF4) that is embedded in ORF1 has recently been identified in the genomes of HEV genotype 1 only [5]. HEV belongs to the family *Hepeviridae*, which is divided into two distinct subfamilies: *Orthohepevirinae* (includes all mammalian and avian HEV) and *Parahepevirinae* (fish HEV) [6]. The *Orthohepevirinae* subfamily is divided into 4 genera (*Paslahepevirus*, *Rocahepevirus*, *Chirohepevirus* and *Avihepevirus*). Members of the genus *Paslahepevirus* have been identified in mammals and are classified into eight genotypes (HEV-1 to 8) [6]. HEV-1 and 2 are responsible for significant waterborne outbreaks in low- and middle-income countries [1, 7]. They infect only humans and are transmitted oro-fecally through consumption of contaminated water [1, 7]. HEV-3 and 4 are responsible for sporadic autochthonous infections in developed countries [1, 7] and infect a wide range of mammals, including pigs, deer, and rabbits. Human infection with HEV-3 and 4 occurs through the consumption of raw or undercooked animal products [1, 7]. HEV-5 and 6 have been identified in feral pigs in Asia; HEV-7 and 8 have been identified in dromedaries and Bactrian camels in countries of the Middle Eastern countries and Asia, respectively [8, 9].

In most developing countries, such as Cameroon, HEV infection in humans is usually occurs via the fecal-oral route, with HEV-1 and 2 being the predominant genotypes. HEV is not a notifiable disease, but a few outbreaks have been reported in Cameroon, such as the 2013 epidemics in the North region of the country. Interestingly, the disease was initially suspected by local health authorities to be a yellow fever (YF) outbreak due to the predominance of jaundice. However, laboratory analysis ruled out yellow fever and identified HEV in 90% of cases, with genotypes 1, 2 and 3 present [10, 11]. Since this outbreak, no other HEV outbreak has been reported in the country and little is known about the prevalence and genetic diversity of the HEV among symptomatic patients in Cameroon.

Since 2004, Cameroon has implemented a national yellow fever (YF) surveillance program in which samples from suspected YF cases are collected and sent to the national yellow fever reference laboratory, the *Centre Pasteur du Cameroun* in Yaoundé. According to the WHO standard definition, a suspected case of YF is an acutely febrile patient who develops jaundice within two weeks of illness onset [12]. Each year, more than 90% of the samples included in this surveillance system are negative for anti-YFV antibodies and/or YFV RNA [13]. Although acute febrile jaundice also suggests infection with a hepatitis virus, these samples are not tested for hepatitis viruses. Therefore, this study aimed to determine the prevalence (anti-HEV antibodies and HEV RNA) of HEV and the diversity of HEV genotypes in yellow fever-negative patients in two major livestock production regions of Cameroon, the Far North (cattle, sheep and goats) and West regions (pigs) [14].

## Materials and methods

### Blood sampling and ELISA

Plasma and serum samples from patients suspected of having yellow fever are routinely transported to *Centre Pasteur du Cameroun* for laboratory confirmation of yellow fever. Remaining samples are stored at -20°C upon completion of testing. Archived yellow fever negative plasma/serum samples collected from yellow fever suspect patients in the Far North and West regions of Cameroon between January 2021 and January 2023 were used for analysis in July 2023. This study was approved by the Institutional Ethics Committee for Research on Human Health of the University of Douala (N °3668 IEC-UDo/06/2023/M). The study was in accordance with the tenets of the Declaration of Helsinki. The samples and database used in this study were pseudonymized by yellow fever surveillance before use, and all precautions were taken to avoid any risk of patient identification. As our study is a retrospective study using samples and a database without identifiable information, informed consent was not required.

The samples were tested for anti-HEV IgM and IgG using a commercially available enzyme-linked immunosorbent assays (ELISA) kits (DIA.PRO Diagnostic Bioprobes) according to the manufacturer’s instructions. Briefly, diluted serum/plasma samples were added to the wells of microtiter plates coated with recombinant HEV-specific antigens. After an initial incubation at +37°C for 60 min, followed by washing, the horseradish peroxidase-conjugated goat anti-human IgM and IgG were added for IgM and IgG detection respectively. This step was followed by the addition of a substrate solution (3, 3, 5, 5,-tetramethylbenzidine) and a second incubation at +37°C for 60 min. After the addition of a stop solution (sulphuric acid) and a third incubation (15 min for IgG detection and 20 min for IgM detection) at room temperature, the presence of HEV IgM or IgG antibodies was indicated by the appearance of a blue colour which changed to yellow. Absorbance was measured at 450 nm using a Multiskan FC spectrophotometer (Thermo Scientific). For each experiment, a blank, a calibrator (for IgG detection only and in duplicate), a negative control (in duplicate for IgM detection and in triplicate for IgG detection) and a positive control (in single for both antibodies) provided in the ELISA kit were included. The cut-off (Co) for each test was determined by calculating the mean optical density (OD) of two negative controls + 0.250 (for IgM detection) and the mean optical density (OD) of three negative controls + 0.350 (for IgG detection). A sample was considered positive if its sample OD/Cuff-off (Co) ratio was >1.2 (for IgM detection) and >1.1 (for IgG detection). A sample was considered negative if its sample OD/Cuff-off (Co) ratio was <1.0 (for IgM detection) and <0.9 (for IgG detection). A sample was considered indeterminate if its sample OD/Cuff-off (Co) ratio was between 1.0–1.2 (for IgM detection) and between 0.9–1.2 (for IgG detection).

### Molecular analyses

#### RNA extraction and HEV detection by real-time RT-PCR

Only ELISA-positive samples (anti-HEV IgM and/or IgG positive samples) collected between January 2022 and January 2023 were selected for molecular testing. Following the manufacturer’s instructions, viral RNA was extracted from 140 μl of these plasma/serum samples using QIAamp Viral RNA Mini Kit (Qiagen, Hilden, Germany). HEV detection was performed according to the protocol developed by Frías *et al*., 2021 [15]. The real‐time RT‐PCR system (Applied Biosystems^®^ QuantStudio^™^ 7 Flex Real-Time PCR System, city country) and SuperScript^™^ III One-Step qRT-PCR System with Platinum Taq (Life Technologies, city, state, USA) were used. Briefly, 20 μM of forward (HEV5260: 5′-RGTRGTTTCTGGGGTGAC-3′) and reverse (HEV5330:5′-AKGGRTTGGTTGGRTGAA-3′) primers and 15 μM of probe (HEV5283: 5′-FAM-TGAYTCYCARCCCTTCGC-TAMRA-3′) were used. The cDNA was synthetized at 50°C for 30 min followed by 95°C for 15 min; then the amplification and fluorescence quantification were performed during 45 cycles of 94°C for 1 min, 51°C for 1 min, and 72°C for 1 min. The experiment was validated if the positive control showed a sigmoidal curve with Ct < 37 and the negative control did not.

#### HEV amplification by nested RT-PCR, sequencing and phylogenetic analyses

All real-time RT-PCR positive samples were subjected to a nested RT-PCR amplification targeting the ORF2 using the Gene Amp PCR System 9700 (manufacturer, city, country) according to the protocol developed by Frías *et al*. in 2021 [15]. The RT-PCR was performed using the SuperScript^™^ III One-Step RT-PCR System with Platinum Taq (Invitrogen by Life Technologies, city, state USA). Briefly, 10 μL of total RNA was added to a reaction mixture containing 0.2 μM of each primer (forward primer HEV-5920S: 5′-CAAGGHTGGCGYTCKGTTGAGAC-3′ and reverse primer HEV-6425A: 5′-CCCTTRTCCTGCTGAGCRTTCTC-3′), 12.3mM MgSO4 and 200μM dNTPs. Amplification started with cDNA synthesis for 30 min at 50°C and an initial denaturation for 2 min at 94°C followed by 40 PCR cycles of 15 sec at 94°C, 30 sec at 60°C and 1 min at 72°C and a final extension for 5 min at 72°C. For the nested PCR, 5 μL of RT-PCR products were used in a final volume of 50 μL containing (Life Technologies Corporation, USA), and 0.2 μM of each primer (forward primer HEV-5930S: 5′-GYTCKGTTGAGACCWCBGGBGT-3′ and reverse primer HEV-6334A: 5′-TTMACWGTCRGCTCGCCATTGGC-3′). Amplification started with an initial denaturation of 5 min at 94°C followed by 40 PCR cycles of 30 sec at 94°C, 30 sec at 55°C, 1 min 30 sec at 72°C, and final extension of 10 min at 72°C. Amplicons (~467bp) were visualized on 2% agarose gel electrophorese and bidirectional sequencing (forward and reverse) was performed using the BigDye Terminator technology (Inqaba Biotec, Pretoria, South Africa). Consensus sequences were obtained after manual editing of forward and reverse sequences using CLC Main Workbench software (Version 5.5.2). HEV genotype/subtype assignment was performed using the HEVnet genotyping tool (https://www.rivm.nl/mpf/typingtool/hev/) and confirmed by BLAST [16]. The reference sequences for the subtypes were described by Smith *et al*., 2016 [17]. Once the reference sequences were obtained, phylogenetic trees were constructed using the neighbour-joining method with MEGA software (Version 7). Genetic distances were calculated using the Kimura two-parameter model as a correction factor and 1000 replicates.

### Statistical analysis

Patient information including age, sex, region of collection, district of collection and date of symptom onset were extracted from the yellow fever surveillance database. Statistical analyses were performed with SPSS 16.0 software. A *p*-value ≤0.05 was considered statistically significant. Prevalences are presented as percentages and categorical variables were compared using chi-squared tests.

## Results

### Prevalence of anti-HEV antibodies

In total, 543 samples were tested for anti-HEV antibodies and 121 samples (22.3%, 95% CI: 19.0–26.0%) were positive for at least one serologic marker of HEV infection. Of these, 8.1% (44/543) were positives for anti-HEV IgM, 5.9% (32/543) for anti-HEV IgG, and 8.3% (45/544) for both markers (Table 1).

**Table 1:** Results of detection of specific anti-hepatitis E virus antibodies.

	Far North n (%)	West n (%)	Total n (%)
**Anti-IgM and/or IgG positive**	99 (27.1)	22 (12,4)	121 (22.3)
**Anti-IgM positive and IgG negative**	26 (7.1)	18 (10.2)	44 (8.1)
**Anti-IgG positive and IgM negative**	30 (8.2)	2 (1.1)	32 (5.9)
**Anti-IgM and IgG positives**	43 (11.8)	2 (1.1)	45 (8.3)
**Negative**	267 (72.7)	155 (88.1)	422 (77.7)
**Total**	366	177	543

IgG: Immunoglobulin G, IgM: Immunoglobulin M, N.: number

Of the 366 samples collected in the Far North region and 177 samples collected in the West region, 99 (27.1%) and 22 (12.4%) respectively, were positive for at least one serological marker. In the Far North region, the positivity rates for IgM, IgG and both markers were 7.1% (26/366), 8.2% (30/366), and 12.0% (43/366), respectively. In the West region, the IgM positivity rate was 10.2% (18/177), while 1.1% (3/177) were seropositive for anti-HEV IgG and 1.1% (2/177) had both serological markers. Statistical analysis showed a significant association between seroprevalence, patient age and region of sample collection (<0.05) (Table 2).

**Table 2:** Seroprevalence of HEV infection by socio-demographic factors.

	N	IgM positives, n (%)	P-value	IgG positives, n (%)	P-value	IgG and IgM positives, n (%)	P-value
**Gender**							
Male	320	26 (8.1)	0.9	18 (5.6)	0.7	26 (8.1)	0.8
Female	223	18 (8.1)		14 (6.3)		19 (8.5)	
**Age (Year)**							
[0-15]	295	16 (5.4)	<0.001	10 (3.4)	<0.001	6 (2.0)	<0.001
]15-30]	146	17 (11.6)		11 (7.5)		18 (12.3)	
]30-45]	73	8 (11.0)		5 (6.9)		18 (24.7)	
]45-60]	22	2 (9.1)		4 (18.2)		1 (4.6)	
> 60	7	1 (14.3)		2 (28.6)		2 (28.6)	
**Year** ^a^							
2021	179	13 (7.3)	0.8	15 (8.4)	0.4	14 (7.8)	0.7
2022	357	30 (8.4)		17 (4.8)		31 (8.7)	
**Region** ^b^							
Far North	187	13 (7.0)	0.007	15 (8.0)	<0.001	29 (15.5)	<0.001
West	177	18 (10.2)		2 (1.1)		2 (1.1)	

IgG: Immunoglobulin G, IgM: Immunoglobulin M, n: Number positive, N: Number tested.

^a^ We did not include 2023 because samples were only collected in January.

^b^ We have not included the results for 2021, as this year samples analyzed were collected only in the Far North region.

### Prevalence of HEV RNA, genotyping and phylogenetic analysis of HEV

**Fig 1:**
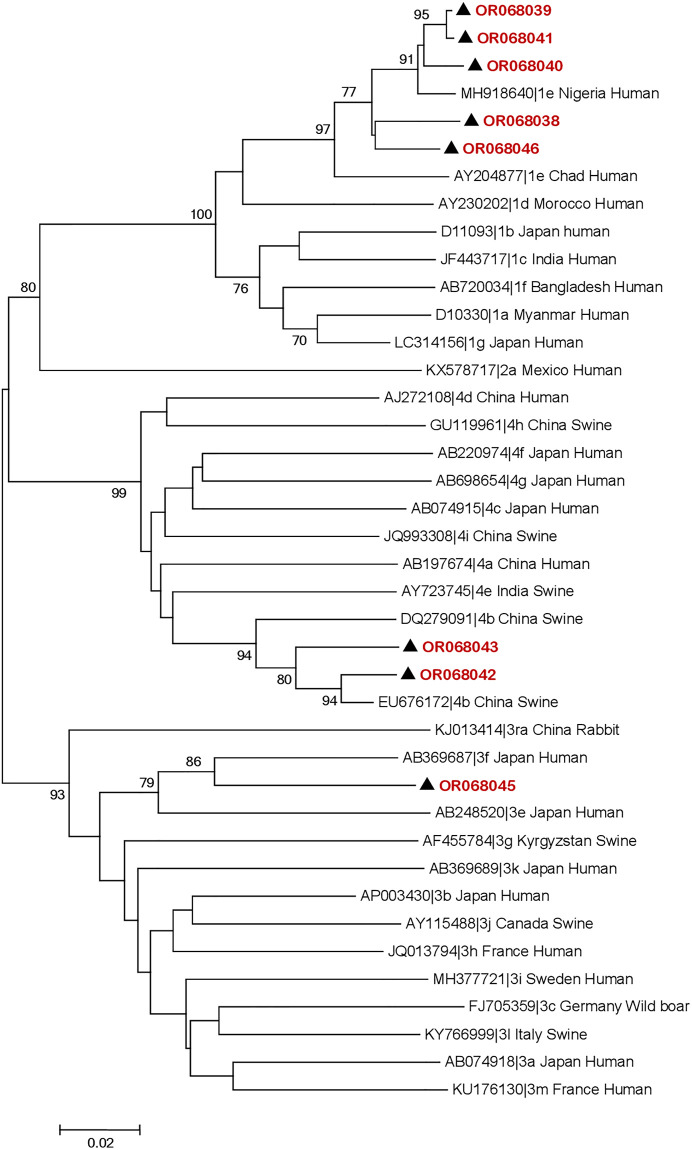
Phylogenetic tree based on a fragment of the ORF2 region of GenBank sequences. Subtyping classification according to Smith et al. (2016) proposed standard HEV strains for subtyping. Only bootstrap values > 70% are presented. The accession number, genotype/subtype, country of origin and host are shown for each GenBank HEV strain used in the phylogenetic analysis. The strains identified in this study are indicated by ▲, coloured red and accession number.

**Fig 2:**
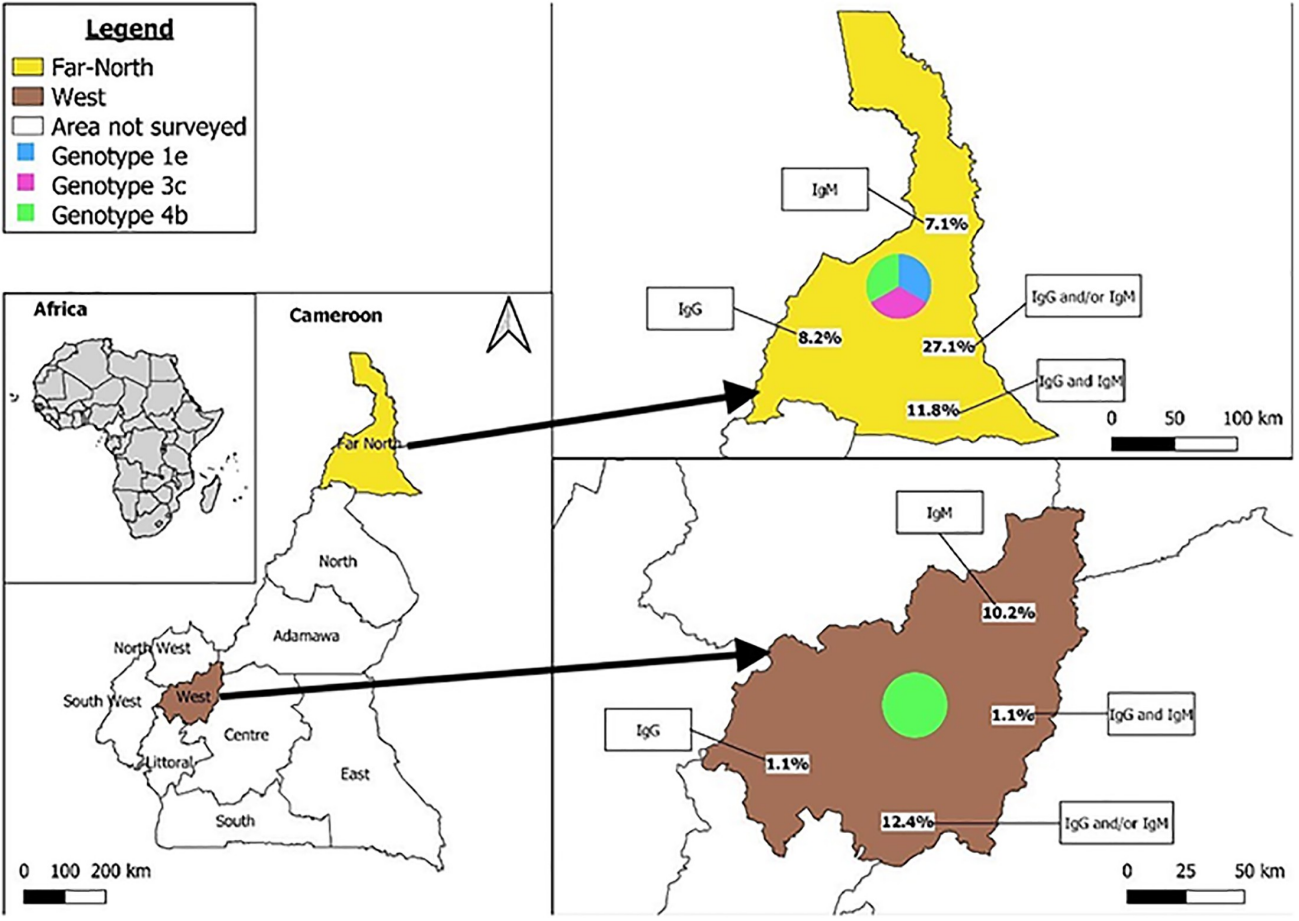
Map of Cameroon showing the Far North and West regions. HEV genotypes for each region and the seroprevalence of HEV (anti-HEV IgM and/or IgG; anti-IgM; anti-IgG; both markers) in YF-negative patients.

HEV RNA was detected in 15.2% (12/79) of the samples tested, with 11 in the Far North region and 1 in the West region. Of these real-time RT-PCR-positive samples, eight (7 in the Far North region and 1 in the West region) were amplified by nested RT-PCR. The nucleotide sequences of HEV strains isolated in this study have been deposited in GenBank (accession numbers: OR068038-OR068046). Phylogenetic analysis of the eight HEV isolates was performed by comparing a 467 bp region of ORF2 with HEV strains from different geographical regions contained in GenBank. Phylogenetic analysis showed that there were three distinct genotypes/subtypes. Five sequences clustered with genotype 1, subtype 1e were grouped together with human HEV strains from African countries such as Nigeria and Chad. Two sequences clustered with genotype 4, subtype 4b and were grouped with strains isolated from pigs in China and India and with human strains isolated in Japan. One strain belonged to genotype 3, subtype 3f and clustered with human HEV strains from Japan. Genotypes 1 (subtype 1e), 3 (subtype 3f) and 4 (subtype 4b) were identified in the Far North region, while only genotype 4 (subtype 4b) was identified in the West region (Figs 1 and 2).

## Discussions

In this study we describe the prevalence and genotypes of HEV in yellow fever suspect patients with acute febrile jaundice in the Far North and West regions of Cameroon. We showed that hepatitis E virus is one of the causes of acute febrile jaundice in patients included in the yellow fever surveillance program in Cameroon. We obtained an overall seroprevalence (22.3%) that was lower than those obtained in 2013 in the North region (89.2%) and in 2019 in the South and Center regions of Cameroon (31.3%) [10, 18]. These differences may be due to different epidemiological settings in the 2013 study and the status of the patients included in the 2019 study. Indeed, the 2013 study detected the first HEV outbreak in Cameroon, while the 2019 study focused on people at risk of developing severe forms of HEV infection, i.e. pregnant women, the elderly and HIV patients. Available African studies show variable prevalences of HEV in the YF-negative patients [19–22]. Our seroprevalence is similar to that found in the Central African Republic (26%), higher than in Burkina Faso (2.6%) and the Democratic Republic of Congo (10.4%), but lower than that found in Chad (34.1%). Only the Central African Republic study used the same kit as this study (ELISA Dia.Pro, Diagnostic Bioprobes srl, Milan, Italy). Comparative studies have shown that the performance of HEV ELISA kits is variable [23]. As there is no gold standard HEV ELISA kit for screening anti-HEV antibodies, comparisons between seroprevalence studies are difficult and should be interpreted with caution [24].

Our study reports for the second time the detection and characterization of HEV RNA in humans in Cameroon. HEV RNA was first detected and characterized in 2020 in a retrospective study that confirmed serological data from the first HEV outbreak which occurred in 2013 [11]. Two HEV genotypes circulating during this outbreak were identified, namely genotypes 1 (subtype 1e) and 3 (subtype 3f). In addition to these two previously reported genotypes, we described HEV genotype 4, subtype 4b, for the first time in humans in Cameroon. To the best of our knowledge, this is the first description of this zoonotic HEV genotype in humans in Africa. This HEV genotype 4, subtype 4b was detected in two different locations, in the West and Far North regions of Cameroon, where pigs, the main reservoir of this genotype, are intensively reared. It should be noted that HEV genotype 4 has also been detected in wild boar, another Suidae species [25]. Wild boar are present in Cameroon, but no HEV studies have been conducted to identify the HEV genotype circulating in this animal population. Such studies would perhaps help to understand the presence of HEV genotype 4 in these two regions of Cameroon. The presence of HEV genotype 4, in addition to the previously described HEV genotype 3 in humans, pigs and wastewater in Cameroon, supports and strengthens the hypothesis of zoonotic transmission of HEV in Cameroon [11, 26, 27]. Importation of pigs from European and Asian countries could be the route of introduction of HEV genotypes 3 and 4 into Cameroon [28]. Understanding the epidemiology of HEV in African countries such as Cameroon will facilitate the implementation of evidence-based control strategies to prevent the spread of HEV. We identified HEV subtypes 1e, 3f and 4b in the Far North region, but only HEV subtype 4b in the West region. This may be explained by the geographical location of the two regions. Administratively, Cameroon is divided into 10 regions with two climatic zones (Fig 2). Northern Cameroon comprises 3 regions, including the Far North region with a tropical climate and Southern Cameroon comprises 7 regions, including the West region with an equatorial climate [29, 30]. The Far North region borders the North, where the first HEV outbreak was reported in 2013, with HEV genotypes 1 and 3 identified [11]. Our recent study of wastewater from the 10 administrative regions of Cameroon reported the presence of HEV genotype 3 in wastewater from the North region [27]. The Far North borders Chad, Niger and Nigeria, where outbreaks of HEV genotype 1 have been reported in recent years [31, 32]. All these data suggest the active circulation of multiple HEV genotypes in the Northern part of Cameroon. The presence of HEV genotype 4 in Southern Cameroon and in humans is hypothetical. In fact, this is the first time that HEV has been identified in humans in this part of the country. Studies in pigs, the main reservoirs of this genotype, have all shown the presence of genotype 3. Further studies, particularly on the likely source, are needed to address some of the unanswered questions.

Our study highlights the need to include HEV as a differential diagnosis in suspected cases of YF and provide clear evidence of the power of integrated disease surveillance. We believe that future larger-scale studies will certainly provide clear answers to some of the unanswered questions about the epidemiological situation of HEV in Cameroon. Indeed, it will be important to elucidate the routes used to transmit zoonotic HEV genotypes to humans in Cameroon and the extent of HEV genotype 4 coverage. It should be noted that similar studies carried out in a number of sub-Saharan African countries have reported, in addition to HEV, significant proportions of other hepatitic viruses including HAV, HBV and HCV [22, 33]. Our study was limited to HEV, but future studies will enable us to assess the proportion of other hepatitic viruses in patients with acute febrile jaundice included in the yellow fever surveillance program in Cameroon.

The limitation of this study is that we did not analyze by molecular assays the serologically positive samples collected in 2021 due to the poor storage of these samples. The use of these data would have provided more information on the epidemiology of HEV in patients with acute jaundice in Cameroon.

## Conclusion

Our results showed that hepatitis E virus is one of the causes of acute febrile jaundice in patients included in the YF surveillance program in Cameroon. Our study provides an example of how data from the national YF surveillance program can be used to investigate another disease with a significant impact on the population. We described the circulation of three HEV genotypes, including two zoonotic genotypes. Therefore, further studies will be important to elucidate the transmission routes of these zoonotic HEV genotypes to humans in Cameroon.

## Supporting information

S1 Dataset (PDF)
