# Supplementary material for: Hepatitis E virus infections among patients with acute febrile jaundice in two regions of Cameroon: First molecular characterization of hepatitis E virus genotype 4
Source: PLoS One. 2024 Feb 12;19(2):e0298723. doi: 10.1371/journal.pone.0298723 (PMC10861035; doi:10.1371/journal.pone.0298723)
Supplement: S1 Dataset — (PDF) [file pone.0298723.s001.pdf]

Region	Age range	Sex	Date of Onset	Year of sampling	IgM results	IgG results	IgM and IgG results	IgM and/or IgG results	qRT-PCR results	Genotypes
West	1	F	janv.-22	2022	Negatif	Negatif	Negatif	Negatif	NA	
Far North	1	M	janv.-22	2022	Negatif	Negatif	Negatif	Negatif	NA	
West	2	M	janv.-22	2022	Negatif	Negatif	Negatif	Negatif	NA	
Far North	1	M	janv.-22	2022	Negatif	Negatif	Negatif	Negatif	NA	
Far North	2	M	janv.-22	2022	Negatif	Negatif	Negatif	Negatif	NA	
West	1	M	janv.-22	2022	Negatif	Negatif	Negatif	Negatif	NA	
West	2	M	janv.-22	2022	Negatif	Negatif	Negatif	Negatif	NA	
Far North	1	F	janv.-22	2022	**Positif**	**Positif**	**Positif**	**Positif**	**Positif**	
Far North	1	M	janv.-22	2022	Negatif	**Positif**	Negatif	**Positif**	Negatif	
Far North	1	M	janv.-22	2022	Negatif	Negatif	Negatif	Negatif	NA	
Far North	2	F	janv.-22	2022	Negatif	Negatif	Negatif	Negatif	NA	
Far North	3	F	janv.-22	2022	Negatif	Negatif	Negatif	Negatif	NA	
Far North	1	M	janv.-22	2022	Negatif	Negatif	Negatif	Negatif	NA	
Far North	2	F	janv.-22	2022	**Positif**	**Positif**	**Positif**	**Positif**	**Positif**	
Far North	2	M	févr.-22	2022	Negatif	**Positif**	Negatif	**Positif**	Negatif	
Far North	2	M	janv.-22	2022	Negatif	Negatif	Negatif	Negatif	NA	
Far North	1	M	janv.-22	2022	Negatif	Negatif	Negatif	Negatif	NA	
Far North	1	F	janv.-22	2022	Negatif	Negatif	Negatif	Negatif	NA	
Far North	1	F	févr.-22	2022	**Positif**	Negatif	Negatif	**Positif**	Negatif	
West	3	F	janv.-22	2022	Negatif	Negatif	Negatif	Negatif	NA	
Far North	1	F	févr.-22	2022	Negatif	Negatif	Negatif	Negatif	NA	
West	1	F	févr.-22	2022	Negatif	Negatif	Negatif	Negatif	NA	
Far North	2	F	févr.-22	2022	Negatif	**Positif**	Negatif	**Positif**	Negatif	

Far North	2	F	févr.-22	2022	**Positif**	**Positif**	**Positif**	**Positif**	**Positif**	1e
West	1	F	févr.-22	2022	Negatif	Negatif	Negatif	Negatif		
West	3	F	févr.-22	2022	**Positif**	Negatif	Negatif	**Positif**	Negatif	
Far North	1	F	févr.-22	2022	Negatif	Negatif	Negatif	Negatif	NA	
West	1	M	févr.-22	2022	Negatif	Negatif	Negatif	Negatif	NA	
West	1	M	févr.-22	2022	Negatif	Negatif	Negatif	Negatif	NA	
Far North	2	M	févr.-22	2022	Negatif	Negatif	Negatif	Negatif	NA	
Far North	2	M	janv.-22	2022	**Positif**	**Positif**	**Positif**	**Positif**	Negatif	
Far North	1	M	mars-22	2022	Negatif	Negatif	Negatif	Negatif	NA	
Far North	2	M	mars-22	2022	Negatif	Negatif	Negatif	Negatif	NA	
Far North	1	M	mars-22	2022	Negatif	Negatif	Negatif	Negatif	NA	
Far North	1	F	mars-22	2022	Negatif	Negatif	Negatif	Negatif	NA	
Far North	1	M	mars-22	2022	Negatif	Negatif	Negatif	Negatif	NA	
Far North	4	M	mars-22	2022	Negatif	**Positif**	Negatif	**Positif**	Negatif	
Far North	5	F	mars-22	2022	Negatif	**Positif**	Negatif	**Positif**	Negatif	
Far North	1	M	mars-22	2022	Negatif	Negatif	Negatif	Negatif	NA	
Far North	1	M	févr.-22	2022	Negatif	Negatif	Negatif	Negatif	NA	
Far North	1	F	nov.-22	2022	Negatif	Negatif	Negatif	Negatif	NA	
West	4	M	mars-22	2022	Negatif	Negatif	Negatif	Negatif	NA	
West	1	F	mars-22	2022	**Positif**	Negatif	Negatif	**Positif**	Negatif	
Far North	1	M	mars-22	2022	**Positif**	Negatif	Negatif	**Positif**	Negatif	
Far North	1	M	mars-22	2022	Negatif	Negatif	Negatif	Negatif	NA	
Far North	1	M	mars-22	2022	Negatif	Negatif	Negatif	Negatif	NA	
West	1	M	mars-22	2022	Negatif	Negatif	Negatif	Negatif	NA	
West	4	M	mars-22	2022	Negatif	Negatif	Negatif	Negatif	NA	
West	1	F	mars-22	2022	Negatif	Negatif	Negatif	Negatif	NA	

Far North	1	F	mars-22	2022	Negatif	Negatif	Negatif	Negatif	NA	
Far North	1	F	mars-22	2022	Negatif	Negatif	Negatif	Negatif	NA	
Far North	2	M	mars-22	2022	Negatif	Negatif	Negatif	Negatif	NA	
Far North	2	M	mars-22	2022	Negatif	**Positif**	Negatif	**Positif**	Negatif	
Far North	1	M	mars-22	2022	Negatif	Negatif	Negatif	Negatif	NA	
Far North	1	M	mars-22	2022	Negatif	Negatif	Negatif	Negatif	NA	
West	2	F	mars-22	2022	**Positif**	Negatif	Negatif	**Positif**	Negatif	
Far North	4	F	mars-22	2022	Negatif	Negatif	Negatif	Negatif	NA	
West	1	F	mars-22	2022	**Positif**	Negatif	Negatif	**Positif**	Negatif	
Far North	4	M	mars-22	2022	Negatif	Negatif	Negatif	Negatif	NA	
Far North	3	F	mars-22	2022	**Positif**	**Positif**	**Positif**	**Positif**	**Positif**	1e
Far North	3	M	mars-22	2022	**Positif**	**Positif**	**Positif**	**Positif**	Negatif	
Far North	3	M	mars-22	2022	Negatif	Negatif	Negatif	Negatif	NA	
West	1	M	mars-22	2022	Negatif	Negatif	Negatif	Negatif	NA	
West	1	M	mars-22	2022	Negatif	Negatif	Negatif	Negatif	NA	
West	2	M	mars-22	2022	Negatif	Negatif	Negatif	Negatif	NA	
Far North	1	M	mars-22	2022	Negatif	Negatif	Negatif	Negatif	NA	
Far North	3	F	mars-22	2022	**Positif**	**Positif**	**Positif**	**Positif**	**Positif**	1e
Far North	1	M	avr.-22	2022	Negatif	**Positif**	Negatif	**Positif**	Negatif	
West	1	F	avr.-22	2022	Negatif	Negatif	Negatif	Negatif	NA	
West	2	M	avr.-22	2022	**Positif**	Negatif	Negatif	**Positif**	Negatif	
West	1	F	avr.-22	2022	Negatif	Negatif	Negatif	Negatif	NA	
West	1	F	avr.-22	2022	Negatif	Negatif	Negatif	Negatif	NA	
West	1	F	avr.-22	2022	Negatif	Negatif	Negatif	Negatif	NA	
West	1	M	avr.-22	2022	Negatif	Negatif	Negatif	Negatif	NA	
Far North	2	F	avr.-22	2022	Negatif	Negatif	Negatif	Negatif	NA	
West	1	F	avr.-22	2022	Negatif	Negatif	Negatif	Negatif	NA	
West	1	M	avr.-22	2022	Negatif	Negatif	Negatif	Negatif	NA	

West	3	F	avr.-22	2022	Negatif	Negatif	Negatif	Negatif	NA	
Far North	2	M	avr.-22	2022	Negatif	Negatif	Negatif	Negatif	NA	
West	1	M	avr.-22	2022	Negatif	Negatif	Negatif	Negatif	NA	
West	2	F	avr.-22	2022	Negatif	Negatif	Negatif	Negatif	NA	
West	2	F	avr.-22	2022	Negatif	Negatif	Negatif	Negatif	NA	
West	5	F	avr.-22	2022	Negatif	Negatif	Negatif	Negatif	NA	
West	2	F	avr.-22	2022	Negatif	Negatif	Negatif	Negatif	NA	
West	1	M	avr.-22	2022	Negatif	Negatif	Negatif	Negatif	NA	
West	1	F	avr.-22	2022	Negatif	Negatif	Negatif	Negatif	NA	
Far North	2	F	avr.-22	2022	Negatif	Negatif	Negatif	Negatif	NA	
West	4	F	avr.-22	2022	Negatif	Negatif	Negatif	Negatif	NA	
West	2	F	avr.-22	2022	Negatif	Negatif	Negatif	Negatif	NA	
West	4	F	avr.-22	2022	Negatif	Negatif	Negatif	Negatif	NA	
West	1	M	avr.-22	2022	Negatif	Negatif	Negatif	Negatif	NA	
West	1	F	avr.-22	2022	Negatif	Negatif	Negatif	Negatif	NA	
West	3	M	avr.-22	2022	Negatif	Negatif	Negatif	Negatif	NA	
West	1	M	avr.-22	2022	Negatif	Negatif	Negatif	Negatif	NA	
Far North	2	M	avr.-22	2022	**Positif**	**Positif**	**Positif**	**Positif**	Negatif	
West	2	M	avr.-22	2022	Negatif	Negatif	Negatif	Negatif	NA	
West	2	M	avr.-22	2022	Negatif	Negatif	Negatif	Negatif	NA	
West	2	M	avr.-22	2022	Negatif	Negatif	Negatif	Negatif	NA	
West	1	M	avr.-22	2022	Negatif	Negatif	Negatif	Negatif	NA	
West	3	F	avr.-22	2022	Negatif	Negatif	Negatif	Negatif	NA	
West	1	M	avr.-22	2022	Negatif	Negatif	Negatif	Negatif	NA	
West	2	F	avr.-22	2022	**Positif**	Negatif	Negatif	**Positif**	Negatif	
Far North	1	M	avr.-22	2022	Negatif	Negatif	Negatif	Negatif	NA	
West	1	M	avr.-22	2022	Negatif	Negatif	Negatif	Negatif	NA	
West	2	M	avr.-22	2022	Negatif	Negatif	Negatif	Negatif	NA	
West	2	M	avr.-22	2022	Negatif	Negatif	Negatif	Negatif	NA	
West	2	M	avr.-22	2022	Negatif	Negatif	Negatif	Negatif	NA	
West	1	F	avr.-22	2022	Negatif	Negatif	Negatif	Negatif	NA	
West	2	M	avr.-22	2022	Negatif	Negatif	Negatif	Negatif	NA	
West	4	F	avr.-22	2022	**Positif**	Negatif	Negatif	**Positif**	Negatif	
West	2	F	mai-22	2022	Negatif	Negatif	Negatif	Negatif	NA	
West	1	F	mai-22	2022	Negatif	Negatif	Negatif	Negatif	NA	
West	1	F	mai-22	2022	Negatif	Negatif	Negatif	Negatif	NA	
Far North	2	M	mai-22	2022	**Positif**	**Positif**	**Positif**	**Positif**	Negatif	

West	1	F	mai-22	2022	Negatif	Negatif	Negatif	Negatif	NA	
West	3	M	mai-22	2022	Negatif	Negatif	Negatif	Negatif	NA	
West	4	M	mai-22	2022	Negatif	Negatif	Negatif	Negatif	NA	
West	1	F	mai-22	2022	Negatif	Negatif	Negatif	Negatif	NA	
West	2	M	mai-22	2022	Negatif	Negatif	Negatif	Negatif	NA	
West	2	M	mai-22	2022	**Positif**	Negatif	Negatif	**Positif**	Negatif	
West	2	M	mai-22	2022	Negatif	Negatif	Negatif	Negatif	NA	
West	2	F	mai-22	2022	Negatif	Negatif	Negatif	Negatif	NA	
West	1	F	mai-22	2022	Negatif	Negatif	Negatif	Negatif	NA	
West	2	M	mai-22	2022	Negatif	Negatif	Negatif	Negatif	NA	
West	1	F	mai-22	2022	Negatif	Negatif	Negatif	Negatif	NA	
West	1	F	mai-22	2022	Negatif	Negatif	Negatif	Negatif	NA	
West	1	M	mai-22	2022	Negatif	Negatif	Negatif	Negatif	NA	
West	1	M	mai-22	2022	Negatif	Negatif	Negatif	Negatif	NA	
West	5	M	mai-22	2022	**Positif**	Negatif	Negatif	**Positif**	Negatif	
Far North	1	M	mai-22	2022	Negatif	Negatif	Negatif	Negatif	NA	
West	2	M	mai-22	2022	Negatif	Negatif	Negatif	Negatif	NA	
West	4	M	mai-22	2022	**Positif**	Negatif	Negatif	**Positif**	Negatif	
Far North	1	M	mai-22	2022	Negatif	Negatif	Negatif	Negatif	NA	
Far North	1	M	mai-22	2022	Negatif	Negatif	Negatif	Negatif	NA	
Far North	3	F	mai-22	2022	Negatif	Negatif	Negatif	Negatif	NA	
West	1	M	mai-22	2022	Negatif	Negatif	Negatif	Negatif	NA	
West	1	M	mai-22	2022	Negatif	Negatif	Negatif	Negatif	NA	
Far North	2	F	mai-22	2022	Negatif	Negatif	Negatif	Negatif	NA	
Far North	2	F	mai-22	2022	Negatif	**Positif**	Negatif	**Positif**	Negatif	
Far North	2	F	mai-22	2022	**Positif**	**Positif**	**Positif**	**Positif**	Negatif	
Far North	3	M	mai-22	2022	**Positif**	Negatif	Negatif	**Positif**	Negatif	
Far North	1	F	mai-22	2022	Negatif	Negatif	Negatif	Negatif	NA	
West	1	M	mai-22	2022	Negatif	Negatif	Negatif	Negatif	NA	
West	2	M	mai-22	2022	Negatif	Negatif	Negatif	Negatif	NA	
Far North	4	F	mai-22	2022	Negatif	Negatif	Negatif	Negatif	NA	
Far North	1	F	mai-22	2022	Negatif	Negatif	Negatif	Negatif	NA	

Far North	2	M	juin-22	2022	Negatif	**Positif**	Negatif	**Positif**	Negatif	
West	1	F	juin-22	2022	Negatif	Negatif	Negatif	Negatif	NA	
West	2	M	juin-22	2022	Negatif	Negatif	Negatif	Negatif	NA	
West	1	M	juin-22	2022	Negatif	Negatif	Negatif	Negatif	NA	
Far North	3	M	juin-22	2022	**Positif**	Negatif	Negatif	**Positif**	Negatif	
West	1	M	juin-22	2022	Negatif	Negatif	Negatif	Negatif	NA	
Far North	2	F	juin-22	2022	**Positif**	**Positif**	**Positif**	**Positif**	**Positif**	1e
West	2	M	juin-22	2022	Negatif	Negatif	Negatif	Negatif	NA	
West	1	M	juin-22	2022	Negatif	Negatif	Negatif	Negatif	NA	
West	1	F	juin-22	2022	Negatif	Negatif	Negatif	Negatif	NA	
West	1	M	juin-22	2022	Negatif	Negatif	Negatif	Negatif	NA	
Far North	1	F	juin-22	2022	Negatif	Negatif	Negatif	Negatif	NA	
West	1	M	juin-22	2022	Negatif	Negatif	Negatif	Negatif	NA	
West	2	M	juin-22	2022	Negatif	Negatif	Negatif	Negatif	NA	
West	1	M	juin-22	2022	Negatif	Negatif	Negatif	Negatif	NA	
West	2	M	juin-22	2022	Negatif	Negatif	Negatif	Negatif	NA	
West	1	M	juin-22	2022	Negatif	Negatif	Negatif	Negatif	NA	
Far North	2	M	juin-22	2022	Negatif	Negatif	Negatif	Negatif	NA	
Far North	2	M	juin-22	2022	Negatif	Negatif	Negatif	Negatif	NA	
Far North	2	F	juin-22	2022	Negatif	Negatif	Negatif	Negatif	NA	
Ouest	1	M	juin-22	2022	Negatif	Negatif	Negatif	Negatif	NA	
Far North	2	F	juin-22	2022	Negatif	Negatif	Negatif	Negatif	NA	
West	1	M	juin-22	2022	Negatif	Negatif	Negatif	Negatif	NA	
West	2	M	juin-22	2022	Negatif	**Positif**	Negatif	**Positif**	Negatif	
West	2	F	juil.-22	2022	Negatif	Negatif	Negatif	Negatif	NA	
Far North	1	F	juil.-22	2022	Negatif	Negatif	Negatif	Negatif	NA	
West	1	M	juil.-22	2022	Negatif	Negatif	Negatif	Negatif	NA	
West	2	M	juil.-22	2022	Negatif	Negatif	Negatif	Negatif	NA	
West	1	M	juil.-22	2022	Negatif	Negatif	Negatif	Negatif	NA	
West	1	F	juil.-22	2022	Negatif	Negatif	Negatif	Negatif	NA	
West	1	F	juil.-22	2022	Negatif	Negatif	Negatif	Negatif	NA	
West	4	F	juil.-22	2022	Negatif	Negatif	Negatif	Negatif	NA	
Far North	3	F	juil.-22	2022	Negatif	Negatif	Negatif	Negatif	NA	

West	1	M	juil.-22	2022	Negatif	Negatif	Negatif	Negatif	NA	
West	1	F	juil.-22	2022	Negatif	Negatif	Negatif	Negatif	NA	
West	1	M	juil.-22	2022	Negatif	Negatif	Negatif	Negatif	NA	
West	1	M	juil.-22	2022	Negatif	Negatif	Negatif	Negatif	NA	
Far North	1	M	juil.-22	2022	Negatif	Negatif	Negatif	Negatif	NA	
West	1	F	juil.-22	2022	Negatif	Negatif	Negatif	Negatif	NA	
West	2	M	juil.-22	2022	Negatif	Negatif	Negatif	Negatif	NA	
West	1	F	juil.-22	2022	Negatif	Negatif	Negatif	Negatif	NA	
West	2	F	juil.-22	2022	Negatif	Negatif	Negatif	Negatif	NA	
West	1	F	juil.-22	2022	Negatif	**Positif**	Negatif	**Positif**	Negatif	
West	5	F	juil.-22	2022	**Positif**	**Positif**	**Positif**	**Positif**	Negatif	
West	2	F	juil.-22	2022	Negatif	Negatif	Negatif	Negatif	NA	
West	2	F	juil.-22	2022	Negatif	Negatif	Negatif	Negatif	NA	
West	2	M	juil.-22	2022	Negatif	Negatif	Negatif	Negatif	NA	
West	1	M	juil.-22	2022	**Positif**	Negatif	Negatif	**Positif**	Negatif	
West	1	M	juil.-22	2022	Negatif	Negatif	Negatif	Negatif	NA	
West	1	F	juil.-22	2022	Negatif	Negatif	Negatif	Negatif	NA	
West	1	M	juil.-22	2022	Negatif	Negatif	Negatif	Negatif	NA	
West	1	F	juil.-22	2022	Negatif	Negatif	Negatif	Negatif	NA	
West	2	M	juil.-22	2022	Negatif	Negatif	Negatif	Negatif	NA	
West	3	M	juil.-22	2022	Negatif	Negatif	Negatif	Negatif	NA	
West	1	M	juil.-22	2022	Negatif	Negatif	Negatif	Negatif	NA	
West	1	F	juil.-22	2022	Negatif	Negatif	Negatif	Negatif	NA	
West	2	M	juil.-22	2022	Negatif	Negatif	Negatif	Negatif	NA	
West	3	M	juil.-22	2022	Negatif	Negatif	Negatif	Negatif	NA	
West	2	M	juil.-22	2022	Negatif	Negatif	Negatif	Negatif	NA	
West	1	M	juil.-22	2022	Negatif	Negatif	Negatif	Negatif	NA	
West	2	M	juil.-22	2022	Negatif	Negatif	Negatif	Negatif	NA	
West	1	M	août-22	2022	Negatif	Negatif	Negatif	Negatif	NA	
West	1	F	août-22	2022	Negatif	Negatif	Negatif	Negatif	NA	
Far North	1	F	juil.-22	2022	**Positif**	**Positif**	**Positif**	**Positif**	**Positif**	
West	2	M	août-22	2022	Negatif	Negatif	Negatif	Negatif	NA	
West	1	M	août-22	2022	Negatif	Negatif	Negatif	Negatif	NA	
Far North	1	M	août-22	2022	Negatif	Negatif	Negatif	Negatif	NA	
Far North	2	M	août-22	2022	**Positif**	**Positif**	**Positif**	**Positif**	**Positif**	4b
West	1	M	août-22	2022	Negatif	Negatif	Negatif	Negatif	NA	
West	1	M	août-22	2022	Negatif	Negatif	Negatif	Negatif	NA	
West	2	M	août-22	2022	**Positif**	Negatif	Negatif	**Positif**	Negatif	

Far North	2	M	août-22	2022	Negatif	Negatif	Negatif	Negatif	NA	
West	2	F	août-22	2022	Negatif	Negatif	Negatif	Negatif	NA	
West	2	M	août-22	2022	Negatif	Negatif	Negatif	Negatif	NA	
West	2	M	août-22	2022	Negatif	Negatif	Negatif	Negatif	NA	
West	4	F	août-22	2022	Negatif	Negatif	Negatif	Negatif	NA	
Far North	1	F	août-22	2022	Negatif	Negatif	Negatif	Negatif	NA	
Far North	4	M	août-22	2022	Negatif	**Positif**	Negatif	**Positif**	Negatif	
Far North	1	F	août-22	2022	Negatif	Negatif	Negatif	Negatif	NA	
Far North	2	M	août-22	2022	**Positif**	Negatif	Negatif	**Positif**	Negatif	
Far North	1	M	août-22	2022	**Positif**	Negatif	Negatif	**Positif**	Negatif	
Far North	1	F	août-22	2022	Negatif	Negatif	Negatif	Negatif	NA	
Far North	1	F	août-22	2022	Negatif	Negatif	Negatif	Negatif	NA	
Far North	1	M	août-22	2022	Negatif	Negatif	Negatif	Negatif	NA	
West	1	M	août-22	2022	Negatif	Negatif	Negatif	Negatif	NA	
West	2	F	août-22	2022	Negatif	Negatif	Negatif	Negatif	NA	
Far North	1	M	août-22	2022	Negatif	Negatif	Negatif	Negatif	NA	
Far North	2	M	août-22	2022	**Positif**	**Positif**	**Positif**	**Positif**	Negatif	
Far North	1	M	août-22	2022	Negatif	Negatif	Negatif	Negatif	NA	
West	3	M	août-22	2022	**Positif**	**Positif**	**Positif**	**Positif**	**Positif**	4b
West	2	M	sept.-22	2022	Negatif	Negatif	Negatif	Negatif	NA	
West	2	M	sept.-22	2022	Negatif	Negatif	Negatif	Negatif	NA	
West	1	F	sept.-22	2022	Negatif	Negatif	Negatif	Negatif	NA	
West	1	M	sept.-22	2022	**Positif**	Negatif	Negatif	**Positif**	Negatif	
West	2	M	sept.-22	2022	Negatif	Negatif	Negatif	Negatif	NA	
Far North	5	M	sept.-22	2022	**Positif**	**Positif**	**Positif**	**Positif**	Negatif	
Far North	1	M	août-22	2022	Negatif	Negatif	Negatif	Negatif	NA	
Far North	1	F	sept.-22	2022	Negatif	Negatif	Negatif	Negatif	NA	

Far North	2	M	sept.-22	2022	**Positif**	**Positif**	**Positif**	**Positif**	Negatif	
Far North	2	M	sept.-22	2022	Negatif	Negatif	Negatif	Negatif	NA	
Far North	1	M	sept.-22	2022	Negatif	Negatif	Negatif	Negatif	NA	
Far North	1	F	sept.-22	2022	Negatif	Negatif	Negatif	Negatif	NA	
West	1	F	sept.-22	2022	Negatif	Negatif	Negatif	Negatif	NA	
Far North	2	F	sept.-22	2022	Negatif	Negatif	Negatif	Negatif	NA	
Far North	3	F	sept.-22	2022	Negatif	Negatif	Negatif	Negatif	NA	
West	2	M	sept.-22	2022	**Positif**	Negatif	Negatif	**Positif**	Negatif	
Far North	1	F	sept.-22	2022	Negatif	Negatif	Negatif	Negatif	NA	
Far North	2	M	sept.-22	2022	**Positif**	Negatif	Negatif	**Positif**	Negatif	
Far North	1	F	sept.-22	2022	Negatif	Negatif	Negatif	Negatif	NA	
Far North	2	M	sept.-22	2022	Negatif	**Positif**	Negatif	**Positif**	Negatif	
West	1	F	sept.-22	2022	Negatif	Negatif	Negatif	Negatif	NA	
West	3	M	sept.-22	2022	Negatif	Negatif	Negatif	Negatif	NA	
West	1	M	sept.-22	2022	Negatif	Negatif	Negatif	Negatif	NA	
Far North	1	F	sept.-22	2022	Negatif	Negatif	Negatif	Negatif	NA	
West	1	M	sept.-22	2022	Negatif	Negatif	Negatif	Negatif	NA	
Far North	1	M	sept.-22	2022	Negatif	Negatif	Negatif	Negatif	NA	
Far North	1	M	sept.-22	2022	**Positif**	Negatif	Negatif	**Positif**	Negatif	
Far North	1	F	sept.-22	2022	Negatif	Negatif	Negatif	Negatif	NA	
Far North	2	M	sept.-22	2022	**Positif**	**Positif**	**Positif**	**Positif**	Negatif	
Far North	1	M	sept.-22	2022	Negatif	Negatif	Negatif	Negatif	NA	
West	5	F	sept.-22	2022	Negatif	Negatif	Negatif	Negatif	NA	
West	1	F	sept.-22	2022	Negatif	Negatif	Negatif	Negatif	NA	
West	1	F	sept.-22	2022	Negatif	Negatif	Negatif	Negatif	NA	
Far North	1	F	sept.-22	2022	Negatif	Negatif	Negatif	Negatif	NA	

Far North	1	F	sept.-22	2022	Negatif	Negatif	Negatif	Negatif	NA	
Far North	1	M	sept.-22	2022	Negatif	Negatif	Negatif	Negatif	NA	
Far North	1	M	sept.-22	2022	Negatif	Negatif	Negatif	Negatif	NA	
Far North	1	M	sept.-22	2022	Negatif	Negatif	Negatif	Negatif	NA	
Far North	1	F	oct.-22	2022	Negatif	Negatif	Negatif	Negatif	NA	
Far North	1	M	oct.-22	2022	**Positif**	Negatif	Negatif	**Positif**	Negatif	
Far North	1	M	sept.-22	2022	Negatif	**Positif**	Negatif	**Positif**	Negatif	
West	2	M	oct.-22	2022	Negatif	Negatif	Negatif	Negatif	NA	
Far North	2	M	oct.-22	2022	Negatif	Negatif	Negatif	Negatif	NA	
Far North	1	M	oct.-22	2022	Negatif	Negatif	Negatif	Negatif	NA	
Far North	2	M	oct.-22	2022	**Positif**	**Positif**	**Positif**	**Positif**	**Positif**	1e
Far North	2	M	oct.-22	2022	**Positif**	**Positif**	**Positif**	**Positif**	**Positif**	3c
West	3	M	oct.-22	2022	Negatif	Negatif	Negatif	Negatif	NA	
West	2	F	oct.-22	2022	**Positif**	Negatif	Negatif	**Positif**	Negatif	
Far North	1	M	oct.-22	2022	Negatif	Negatif	Negatif	Negatif	NA	
Far North	1	M	oct.-22	2022	Negatif	Negatif	Negatif	Negatif	NA	
Far North	1	M	oct.-22	2022	Negatif	Negatif	Negatif	Negatif	NA	
Far North	1	M	oct.-22	2022	Negatif	Negatif	Negatif	Negatif	NA	
Far North	4	F	oct.-22	2022	Negatif	**Positif**	Negatif	**Positif**	Negatif	
Far North	2	F	oct.-22	2022	Negatif	Negatif	Negatif	Negatif	NA	
Far North	1	F	oct.-22	2022	Negatif	Negatif	Negatif	Negatif	NA	
Far North	3	F	oct.-22	2022	**Positif**	**Positif**	**Positif**	**Positif**	Negatif	
West	2	F	oct.-22	2022	Negatif	Negatif	Negatif	Negatif	NA	
Far North	1	F	oct.-22	2022	Negatif	Negatif	Negatif	Negatif	NA	

Far North	1	F	oct.-22	2022	Negatif	Negatif	Negatif	Negatif	NA	
Far North	2	F	oct.-22	2022	Negatif	**Positif**	Negatif	**Positif**	Negatif	
Far North	5	F	oct.-22	2022	Negatif	**Positif**	Negatif	**Positif**	Negatif	
Ouest	4	F	oct.-22	2022	Negatif	Negatif	Negatif	Negatif	NA	
Far North	2	F	oct.-22	2022	Negatif	Negatif	Negatif	Negatif	NA	
Far North	2	M	oct.-22	2022	**Positif**	**Positif**	**Positif**	**Positif**	Negatif	
Far North	2	M	oct.-22	2022	Negatif	Negatif	Negatif	Negatif	NA	
Far North	1	F	oct.-22	2022	Negatif	Negatif	Negatif	Negatif	NA	
West	1	M	oct.-22	2022	Negatif	Negatif	Negatif	Negatif	NA	
Far North	1	M	oct.-22	2022	Negatif	Negatif	Negatif	Negatif	NA	
Far North	1	M	oct.-22	2022	**Positif**	**Positif**	**Positif**	**Positif**	Negatif	
Far North	1	M	oct.-22	2022	Negatif	Negatif	Negatif	Negatif	NA	
Far North	1	M	oct.-22	2022	Negatif	Negatif	Negatif	Negatif	NA	
Far North	2	M	oct.-22	2022	**Positif**	Negatif	Negatif	**Positif**	Negatif	
Far North	1	M	nov.-22	2022	Negatif	Negatif	Negatif	Negatif	NA	
Far North	4	M	nov.-22	2022	**Positif**	**Positif**	**Positif**	**Positif**	**Positif**	1e
Far North	2	M	nov.-22	2022	Negatif	Negatif	Negatif	Negatif	NA	
Far North	1	M	nov.-22	2022	Negatif	Negatif	Negatif	Negatif	NA	
Far North	1	M	nov.-22	2022	Negatif	Negatif	Negatif	Negatif	NA	
Far North	2	F	nov.-22	2022	Negatif	Negatif	Negatif	Negatif	NA	
Far North	1	M	nov.-22	2022	Negatif	Negatif	Negatif	Negatif	NA	
West	1	M	nov.-22	2022	**Positif**	Negatif	Negatif	**Positif**	Negatif	
Far North	2	M	nov.-22	2022	**Positif**	Negatif	Negatif	**Positif**	Negatif	

Far North	3	M	nov.-22	2022	Negatif	Negatif	Negatif	Negatif	NA	
West	2	M	nov.-22	2022	Negatif	Negatif	Negatif	Negatif	NA	
West	2	F	nov.-22	2022	Negatif	Negatif	Negatif	Negatif	NA	
Far North	3	F	nov.-22	2022	Negatif	Negatif	Negatif	Negatif	NA	
Far North	1	M	nov.-22	2022	Negatif	Negatif	Negatif	Negatif	NA	
Far North	2	M	nov.-22	2022	Negatif	Negatif	Negatif	Negatif	NA	
Far North	3	F	nov.-22	2022	**Positif**	**Positif**	**Positif**	**Positif**	Negatif	
Far North	1	M	nov.-22	2022	Negatif	Negatif	Negatif	Negatif	NA	
Far North	3	M	nov.-22	2022	Negatif	Negatif	Negatif	Negatif	NA	
Far North	1	M	nov.-22	2022	Negatif	Negatif	Negatif	Negatif	NA	
Far North	1	M	nov.-22	2022	Negatif	Negatif	Negatif	Negatif	NA	
Far North	1	M	nov.-22	2022	Negatif	Negatif	Negatif	Negatif	NA	
Far North	2	F	nov.-22	2022	Negatif	Negatif	Negatif	Negatif	NA	
Far North	1	F	nov.-22	2022	Negatif	Negatif	Negatif	Negatif	NA	
Far North	2	M	nov.-22	2022	**Positif**	**Positif**	**Positif**	**Positif**	Negatif	
Far North	1	M	déc.-22	2022	Negatif	Negatif	Negatif	Negatif	NA	
Far North	1	M	déc.-22	2022	Negatif	Negatif	Negatif	Negatif	NA	
West	2	M	déc.-22	2022	**Positif**	Negatif	Negatif	Positif	Negatif	
West	2	F	déc.-22	2022	Negatif	Negatif	Negatif	Negatif	NA	
Far North	2	M	déc.-22	2022	Negatif	Negatif	Negatif	Negatif	NA	
Far North	1	M	déc.-22	2022	Negatif	Negatif	Negatif	Negatif	NA	
Far North	1	M	déc.-22	2022	Negatif	Negatif	Negatif	Negatif	NA	
Far North	2	M	déc.-22	2022	**Positif**	Negatif	Negatif	Positif	Negatif	
Far North	3	M	déc.-22	2022	**Positif**	**Positif**	**Positif**	**Positif**	Negatif	

West	1	F	déc.-22	2022	Negatif	Negatif	Negatif	Negatif	NA	
West	1	F	déc.-22	2022	Negatif	Negatif	Negatif	Negatif	NA	
Far North	2	F	déc.-22	2022	**Positif**	**Positif**	**Positif**	Positif	Negatif	
Ouest	2	M	déc.-22	2022	Negatif	Negatif	Negatif	Negatif	NA	
Far North	2	M	déc.-22	2022	Negatif	Negatif	Negatif	Negatif	NA	
Far North	1	M	déc.-22	2022	**Positif**	**Positif**	**Positif**	**Positif**	Negatif	
Far North	1	F	déc.-22	2022	**Positif**	**Positif**	**Positif**	**Positif**	Negatif	
Far North	1	M	déc.-22	2022	Negatif	Negatif	Negatif	Negatif	NA	
West	1	F	déc.-22	2022	Negatif	Negatif	Negatif	Negatif	NA	
Far North	1	F	déc.-22	2022	Negatif	Negatif	Negatif	Negatif	NA	
Far North	1	F	déc.-22	2022	Negatif	Negatif	Negatif	Negatif	NA	
Far North	1	F	déc.-22	2022	Negatif	Negatif	Negatif	Negatif	NA	
West	1	M	déc.-22	2022	Negatif	Negatif	Negatif	Negatif	NA	
Far North	1	F	déc.-22	2022	Negatif	Negatif	Negatif	Negatif	NA	
Far North	2	M	déc.-22	2022	**Positif**	Negatif	Negatif	**Positif**	Negatif	
Far North	3	F	déc.-22	2022	Negatif	Negatif	Negatif	Negatif	NA	
Far North	1	M	janv.-23	2023	Negatif	Negatif	Negatif	Negatif	NA	
Far North	3	F	janv.-23	2023	Negatif	Negatif	Negatif	Negatif	NA	
Far North	4	F	janv.-23	2023	Negatif	Negatif	Negatif	Negatif	NA	
Far North	2	F	janv.-23	2023	Negatif	Negatif	Negatif	Negatif	NA	
Far North	3	F	janv.-23	2023	Negatif	Negatif	Negatif	Negatif	NA	
West	2	M	janv.-23	2023	Negatif	Negatif	Negatif	Negatif	NA	
West	1	F	janv.-23	2023	**Positif**	Negatif	Negatif	**Positif**	Negatif	
Far North	1	F	janv.-21	2021	Negatif	Negatif	Negatif	Negatif	NA	
Far North	1	F	janv.-21	2021	Positif	Negatif	Negatif	Positif	NA	

Far North	2	F	janv.-21	2021	Negatif	Negatif	Negatif	Negatif	NA	
Far North	1	M	janv.-21	2021	Negatif	Negatif	Negatif	Negatif	NA	
Far North	2	F	janv.-21	2021	Negatif	Positif	Negatif	Positif	NA	
Far North	2	F	janv.-21	2021	Negatif	Negatif	Negatif	Negatif	NA	
Far North	3	F	janv.-21	2021	Positif	Positif	Positif	Positif	NA	
Far North	1	F	janv.-21	2021	Negatif	Negatif	Negatif	Negatif	NA	
Far North	3	F	janv.-21	2021	Positif	Negatif	Negatif	Positif	NA	
Far North	1	M	févr.-21	2021	Negatif	Negatif	Negatif	Negatif	NA	
Far North	3	M	janv.-21	2021	Negatif	Negatif	Negatif	Negatif	NA	
Far North	1	F	janv.-21	2021	Negatif	Negatif	Negatif	Negatif	NA	
Far North	2	F	févr.-21	2021	Negatif	Positif	Negatif	Positif	NA	
Far North	2	M	févr.-21	2021	Negatif	Negatif	Negatif	Negatif	NA	
Far North	3	M	févr.-21	2021	Positif	Negatif	Negatif	Positif	NA	
Far North	2	M	févr.-21	2021	Negatif	Negatif	Negatif	Negatif	NA	
Far North	1	M	févr.-21	2021	Negatif	Negatif	Negatif	Negatif	NA	
Far North	3	F	févr.-21	2021	Negatif	Negatif	Negatif	Negatif	NA	
Far North	3	M	févr.-21	2021	Negatif	Negatif	Negatif	Negatif	NA	
Far North	4	F	févr.-21	2021	Negatif	Negatif	Negatif	Negatif	NA	
Far North	3	M	févr.-21	2021	Negatif	Positif	Negatif	Positif	NA	
Far North	2	F	févr.-21	2021	Positif	Negatif	Negatif	Positif	NA	
Far North	2	M	févr.-21	2021	Negatif	Negatif	Negatif	Negatif	NA	
Far North	2	F	févr.-21	2021	Negatif	Negatif	Negatif	Negatif	NA	

Far North	1	F	févr.-21	2021	Negatif	Negatif	Negatif	Negatif	NA	
Far North	3	F	févr.-21	2021	Negatif	Negatif	Negatif	Negatif	NA	
Far North	3	M	mars-21	2021	Negatif	Negatif	Negatif	Negatif	NA	
Far North	1	F	févr.-21	2021	Negatif	Negatif	Negatif	Negatif	NA	
Far North	1	M	juin-21	2021	Negatif	Negatif	Negatif	Negatif	NA	
Far North	1	M	juin-21	2021	Negatif	Negatif	Negatif	Negatif	NA	
Far North	1	F	juin-21	2021	Negatif	Negatif	Negatif	Negatif	NA	
Far North	3	F	juin-21	2021	Negatif	Negatif	Negatif	Negatif	NA	
Far North	1	M	juin-21	2021	Negatif	Negatif	Negatif	Negatif	NA	
Far North	1	M	juin-21	2021	Negatif	Negatif	Negatif	Negatif	NA	
Far North	2	M	juin-21	2021	Negatif	Negatif	Negatif	Negatif	NA	
Far North	1	M	juin-21	2021	Negatif	Negatif	Negatif	Negatif	NA	
Far North	1	M	juin-21	2021	Negatif	Negatif	Negatif	Negatif	NA	
Far North	3	F	juin-21	2021	Negatif	Negatif	Negatif	Negatif	NA	
Far North	2	M	mars-21	2021	Negatif	Negatif	Negatif	Negatif	NA	
Far North	1	M	mars-21	2021	Negatif	Negatif	Negatif	Negatif	NA	
Far North	3	F	mars-21	2021	Negatif	Negatif	Negatif	Negatif	NA	
Far North	3	M	mars-21	2021	Negatif	Negatif	Negatif	Negatif	NA	
Far North	3	M	avr.-21	2021	Negatif	Negatif	Negatif	Negatif	NA	
Far North	3	M	avr.-21	2021	Negatif	Negatif	Negatif	Negatif	NA	
Far North	3	F	avr.-21	2021	Negatif	Negatif	Negatif	Negatif	NA	
Far North	3	M	avr.-21	2021	Negatif	Positif	Negatif	Positif	NA	

Far North	1	M	avr.-21	2021	Positif	Negatif	Negatif	Positif	NA	
Far North	1	M	avr.-21	2021	Negatif	Negatif	Negatif	Negatif	NA	
Far North	1	M	mai-21	2021	Negatif	Positif	Negatif	Positif	NA	
Far North	3	F	avr.-21	2021	Positif	Negatif	Negatif	Positif	NA	
Far North	1	F	mai-21	2021	Negatif	Positif	Negatif	Positif	NA	
Far North	3	F	mai-21	2021	Positif	Negatif	Negatif	Positif	NA	
Far North	1	F	mai-21	2021	Negatif	Negatif	Negatif	Negatif	NA	
Far North	4	M	mai-21	2021	Negatif	Negatif	Negatif	Negatif	NA	
Far North	1	M	mai-21	2021	Negatif	Negatif	Negatif	Negatif	NA	
Far North	1	M	mai-21	2021	Negatif	Negatif	Negatif	Negatif	NA	
Far North	3	F	mai-21	2021	Negatif	Negatif	Negatif	Negatif	NA	
Far North	1	M	mai-21	2021	Negatif	Negatif	Negatif	Negatif	NA	
Far North	1	F	mai-21	2021	Negatif	Negatif	Negatif	Negatif	NA	
Far North	1	M	mai-21	2021	Negatif	Negatif	Negatif	Negatif	NA	
Far North	1	M	mai-21	2021	Negatif	Negatif	Negatif	Negatif	NA	
Far North	2	M	mai-21	2021	Negatif	Negatif	Negatif	Negatif	NA	
Far North	2	M	mai-21	2021	Negatif	Negatif	Negatif	Negatif	NA	
Far North	1	M	mai-21	2021	Negatif	Positif	Negatif	Positif	NA	
Far North	1	F	mai-21	2021	Positif	Negatif	Negatif	Positif	NA	
Far North	2	F	juin-21	2021	Negatif	Negatif	Negatif	Negatif	NA	
Far North	3	M	juin-21	2021	Negatif	Negatif	Negatif	Negatif	NA	
Far North	1	F	juin-21	2021	Positif	Negatif	Negatif	Positif	NA	

Far North	1	M	juin-21	2021	Negatif	Negatif	Negatif	Negatif	NA	
Far North	3	F	juin-21	2021	Negatif	Negatif	Negatif	Negatif	NA	
Far North	3	M	juin-21	2021	Negatif	Negatif	Negatif	Negatif	NA	
Far North	1	M	juin-21	2021	Negatif	Negatif	Negatif	Negatif	NA	
Far North	1	M	juil.-21	2021	Negatif	Negatif	Negatif	Negatif	NA	
Far North	1	M	mai-21	2021	Negatif	Negatif	Negatif	Negatif	NA	
Far North	1	M	juin-21	2021	Negatif	Negatif	Negatif	Negatif	NA	
Far North	1	M	juil.-21	2021	Negatif	Negatif	Negatif	Negatif	NA	
Far North	1	F	juin-21	2021	Negatif	Negatif	Negatif	Negatif	NA	
Far North	1	M	juil.-21	2021	Negatif	Negatif	Negatif	Negatif	NA	
Far North	1	F	juil.-21	2021	Negatif	Negatif	Negatif	Negatif	NA	
Far North	3	M	juil.-21	2021	Negatif	Negatif	Negatif	Negatif	NA	
Far North	3	M	juil.-21	2021	Negatif	Positif	Negatif	Positif	NA	
Far North	1	F	juil.-21	2021	Negatif	Negatif	Negatif	Negatif	NA	
Far North	3	F	juil.-21	2021	Negatif	Negatif	Negatif	Negatif	NA	
Far North	3	M	juil.-21	2021	Positif	Positif	Positif	Positif	NA	
Far North	2	M	juil.-21	2021	Negatif	Negatif	Negatif	Negatif	NA	
Far North	3	F	août-21	2021	Positif	Positif	Positif	Positif	NA	
Far North	3	M	août-21	2021	Positif	Positif	Positif	Positif	NA	
Far North	3	F	août-21	2021	Positif	Positif	Positif	Positif	NA	
Far North	3	M	août-21	2021	Positif	Positif	Positif	Positif	NA	
Far North	1	F	août-21	2021	Negatif	Negatif	Negatif	Negatif	NA	

Far North	1	F	août-21	2021	Negatif	Negatif	Negatif	Negatif	NA	
Far North	2	M	août-21	2021	Negatif	Negatif	Negatif	Negatif	NA	
Far North	2	F	août-21	2021	Positif	Negatif	Negatif	Positif	NA	
Far North	1	F	août-21	2021	Negatif	Negatif	Negatif	Negatif	NA	
Far North	1	M	août-21	2021	Negatif	Negatif	Negatif	Negatif	NA	
Far North	2	F	juil.-21	2021	Negatif	Positif	Negatif	Positif	NA	
Far North	1	F	août-21	2021	Negatif	Negatif	Negatif	Negatif	NA	
Far North	3	M	août-21	2021	Positif	Positif	Positif	Positif	NA	
Far North	1	F	août-21	2021	Negatif	Negatif	Negatif	Negatif	NA	
Far North	1	M	août-21	2021	Negatif	Negatif	Negatif	Negatif	NA	
Far North	1	M	sept.-21	2021	Negatif	Negatif	Negatif	Negatif	NA	
Far North	1	F	août-21	2021	Negatif	Negatif	Negatif	Negatif	NA	
Far North	3	F	sept.-21	2021	Negatif	Negatif	Negatif	Negatif	NA	
Far North	2	M	sept.-21	2021	Negatif	Negatif	Negatif	Negatif	NA	
Far North	1	M	août-21	2021	Negatif	Negatif	Negatif	Negatif	NA	
Far North	2	F	sept.-21	2021	Negatif	Negatif	Negatif	Negatif	NA	
Far North	2	M	sept.-21	2021	Positif	Positif	Positif	Positif	NA	
Far North	1	M	sept.-21	2021	Negatif	Negatif	Negatif	Negatif	NA	
Far North	2	F	août-21	2021	Negatif	Negatif	Negatif	Negatif	NA	
Far North	3	F	sept.-21	2021	Positif	Negatif	Negatif	Positif	NA	
Far North	1	F	sept.-21	2021	Negatif	Negatif	Negatif	Negatif	NA	
Far North	1	F	sept.-21	2021	Negatif	Negatif	Negatif	Negatif	NA	

Far North	1	F	sept.-21	2021	Negatif	Negatif	Negatif	Negatif	NA	
Far North	1	M	sept.-21	2021	Negatif	Negatif	Negatif	Negatif	NA	
Far North	1	F	sept.-21	2021	Negatif	Negatif	Negatif	Negatif	NA	
Far North	2	F	sept.-21	2021	Negatif	Negatif	Negatif	Negatif	NA	
Far North	1	M	sept.-21	2021	Negatif	Negatif	Negatif	Negatif	NA	
Far North	1	F	sept.-21	2021	Negatif	Negatif	Negatif	Negatif	NA	
Far North	1	M	sept.-21	2021	Negatif	Negatif	Negatif	Negatif	NA	
Far North	1	M	sept.-21	2021	Negatif	Positif	Negatif	Positif	NA	
Far North	1	M	sept.-21	2021	Negatif	Negatif	Negatif	Negatif	NA	
Far North	2	M	oct.-21	2021	Negatif	Negatif	Negatif	Negatif	NA	
Far North	1	M	août-21	2021	Negatif	Negatif	Negatif	Negatif	NA	
Far North	3	M	oct.-21	2021	Positif	Positif	Positif	Positif	NA	
Far North	1	M	oct.-21	2021	Negatif	Negatif	Negatif	Negatif	NA	
Far North	1	M	oct.-21	2021	Negatif	Negatif	Negatif	Negatif	NA	
Far North	1	M	oct.-21	2021	Negatif	Negatif	Negatif	Negatif	NA	
Far North	2	F	sept.-21	2021	Negatif	Negatif	Negatif	Negatif	NA	
Far North	2	M	sept.-21	2021	Negatif	Negatif	Negatif	Negatif	NA	
Far North	1	M	oct.-21	2021	Negatif	Negatif	Negatif	Negatif	NA	
Far North	2	F	oct.-21	2021	Positif	Positif	Positif	Positif	NA	
Far North	1	F	oct.-21	2021	Negatif	Negatif	Negatif	Negatif	NA	
Far North	1	F	oct.-21	2021	Negatif	Positif	Negatif	Positif	NA	
Far North	1	M	oct.-21	2021	Positif	Negatif	Negatif	Positif	NA	

Far North	1	M	oct.-21	2021	Negatif	Negatif	Negatif	Negatif	NA	
Far North	1	M	oct.-21	2021	Negatif	Negatif	Negatif	Negatif	NA	
Far North	4	M	oct.-21	2021	Negatif	Negatif	Negatif	Negatif	NA	
Far North	1	F	oct.-21	2021	Negatif	Negatif	Negatif	Negatif	NA	
Far North	1	M	oct.-21	2021	Negatif	Negatif	Negatif	Negatif	NA	
Far North	1	M	oct.-21	2021	Negatif	Negatif	Negatif	Negatif	NA	
Far North	1	M	oct.-21	2021	Negatif	Negatif	Negatif	Negatif	NA	
Far North	1	M	oct.-21	2021	Negatif	Negatif	Negatif	Negatif	NA	
Far North	1	M	oct.-21	2021	Negatif	Negatif	Negatif	Negatif	NA	
Far North	1	M	oct.-21	2021	Negatif	Negatif	Negatif	Negatif	NA	
Far North	3	M	oct.-21	2021	Negatif	Negatif	Negatif	Negatif	NA	
Far North	3	F	oct.-21	2021	Negatif	Negatif	Negatif	Negatif	NA	
Far North	1	M	oct.-21	2021	Negatif	Positif	Negatif	Positif	NA	
Far North	1	F	oct.-21	2021	Negatif	Negatif	Negatif	Negatif	NA	
Far North	1	F	oct.-21	2021	Negatif	Negatif	Negatif	Negatif	NA	
Far North	1	M	oct.-21	2021	Negatif	Negatif	Negatif	Negatif	NA	
Far North	1	M	oct.-21	2021	Negatif	Negatif	Negatif	Negatif	NA	
Far North	1	M	oct.-21	2021	Negatif	Negatif	Negatif	Negatif	NA	
Far North	1	M	nov.-21	2021	Negatif	Negatif	Negatif	Negatif	NA	
Far North	1	M	oct.-21	2021	Negatif	Negatif	Negatif	Negatif	NA	
Far North	3	M	oct.-21	2021	Positif	Positif	Positif	Positif	NA	
Far North	3	F	oct.-21	2021	Negatif	Negatif	Negatif	Negatif	NA	

Far North	2	M	nov.-21	2021	Positif	Negatif	Negatif	Positif	NA	
Far North	1	M	nov.-21	2021	Negatif	Negatif	Negatif	Negatif	NA	
Far North	2	F	nov.-21	2021	Negatif	Negatif	Negatif	Negatif	NA	
Far North	3	M	nov.-21	2021	Positif	Positif	Positif	Positif	NA	
Far North	1	F	nov.-21	2021	Positif	Positif	Positif	Positif	NA	
Far North	1	M	oct.-21	2021	Negatif	Negatif	Negatif	Negatif	NA	
Far North	3	F	nov.-21	2021	Negatif	Positif	Negatif	Positif	NA	
Far North	1	M	oct.-21	2021	Negatif	Negatif	Negatif	Negatif	NA	
Far North	1	F	nov.-21	2021	Negatif	Negatif	Negatif	Negatif	NA	
Far North	1	F	nov.-21	2021	Negatif	Negatif	Negatif	Negatif	NA	
Far North	1	M	nov.-21	2021	Negatif	Negatif	Negatif	Negatif	NA	
Far North	1	F	nov.-21	2021	Negatif	Negatif	Negatif	Negatif	NA	
Far North	1	F	nov.-21	2021	Negatif	Negatif	Negatif	Negatif	NA	
Far North	3	F	nov.-21	2021	Positif	Positif	Positif	Positif	NA	
Far North	1	M	nov.-21	2021	Negatif	Negatif	Negatif	Negatif	NA	
Far North	4	F	nov.-21	2021	Negatif	Positif	Negatif	Positif	NA	
Far North	1	F	nov.-21	2021	Negatif	Negatif	Negatif	Negatif	NA	
Far North	1	M	nov.-21	2021	Negatif	Negatif	Negatif	Negatif	NA	
Far North	1	M	nov.-21	2021	Negatif	Negatif	Negatif	Negatif	NA	
Far North	3	M	nov.-21	2021	Negatif	Positif	Negatif	Positif	NA	
Far North	1	M	nov.-21	2021	Negatif	Negatif	Negatif	Negatif	NA	
Far North	1	M	nov.-21	2021	Negatif	Negatif	Negatif	Negatif	NA	

Far North	1	M	nov.-21	2021	Negatif	Negatif	Negatif	Negatif	NA	

NA : Not analyzed ; M : Male ; F : Female

Age range : 1=[0-15]; 2=]15-30]; 3=]30-45]; 4=]45-60]; 5=> 60.
